# Identification of driver copy number alterations in diverse cancer types and application in drug repositioning

**DOI:** 10.1002/1878-0261.12112

**Published:** 2017-08-03

**Authors:** Wenbin Zhou, Zhangxiang Zhao, Ruiping Wang, Yue Han, Chengyu Wang, Fan Yang, Ya Han, Haihai Liang, Lishuang Qi, Chenguang Wang, Zheng Guo, Yunyan Gu

**Affiliations:** ^1^ Department of Systems Biology College of Bioinformatics Science and Technology Harbin Medical University China; ^2^ Training Center for Student Innovation and Entrepreneurship Education Harbin Medical University China; ^3^ Department of Pharmacology Harbin Medical University China; ^4^ Department of Bioinformatics Key Laboratory of Ministry of Education for Gastrointestinal Cancer Fujian Medical University Fuzhou China; ^5^ Fujian Key Laboratory of Tumor Microbiology Fujian Medical University Fuzhou China

**Keywords:** copy number alterations, driver, drug repositioning, lncRNAs

## Abstract

Results from numerous studies suggest an important role for somatic copy number alterations (SCNAs) in cancer progression. Our work aimed to identify the drivers (oncogenes or tumor suppressor genes) that reside in recurrently aberrant genomic regions, including a large number of genes or non‐coding genes, which remain a challenge for decoding the SCNAs involved in carcinogenesis. Here, we propose a new approach to comprehensively identify drivers, using 8740 cancer samples involving 18 cancer types from The Cancer Genome Atlas (TCGA). On average, 84 drivers were revealed for each cancer type, including protein‐coding genes, long non‐coding RNAs (lncRNA) and microRNAs (miRNAs). We demonstrated that the drivers showed significant attributes of cancer genes, and significantly overlapped with known cancer genes, including *MYC*,* CCND1* and *ERBB2* in breast cancer, and the lncRNA *PVT1* in multiple cancer types. Pan‐cancer analyses of drivers revealed specificity and commonality across cancer types, and the non‐coding drivers showed a higher cancer‐type specificity than that of coding drivers. Some cancer types from different tissue origins were found to converge to a high similarity because of the significant overlap of drivers, such as head and neck squamous cell carcinoma (HNSC) and lung squamous cell carcinoma (LUSC). The lncRNA *SOX2‐OT*, a common driver of HNSC and LUSC, showed significant expression correlation with the oncogene *SOX2*. In addition, because some drivers are common in multiple cancer types and have been targeted by known drugs, we found that some drugs could be successfully repositioned, as validated by the datasets of drug response assays in cell lines. Our work reported a new method to comprehensively identify drivers in SCNAs across diverse cancer types, providing a feasible strategy for cancer drug repositioning as well as novel findings regarding cancer‐associated non‐coding RNA discovery.

**Abbreviations**
BH: Benjamini and Hochberg
BLCA: bladder urothelial carcinoma
BRCA: breast invasive carcinoma
CCLE: Cancer Cell Line Encyclopedia
CDEGs: correlated differentially expressed genes
CESC: cervical squamous cell carcinoma and endocervical adenocarcinoma
COAD: colon adenocarcinoma
DEGs: differentially expressed genes
FDA: US Food and Drug Administration
FDR: false discovery rate
GBM: glioblastoma multiforme
GDSC: Genomics of Drug Sensitivity in Cancer
GO: gene ontology
HMDD: Human microRNA Disease Database
HNSC: head and neck squamous cell carcinoma
IC50: half maximal inhibitory concentration
*K*_a_: non‐synomymous substitution rate
KEGG: Kyoto Encyclopedia of Genes and Genomes
KIRC: kidney renal clear cell carcinoma
KIRP: kidney renal papillary cell carcinoma
*K*_s_: synonymous substitution rate
LGG: brain lower grade glioma
LIHC: liver hepatocellular carcinoma
LUAD: lung adenocarcinoma
LUSC: lung squamous cell carcinoma
NSCLC: non‐small cell lung cancer
OV: ovarian serous cystadenocarcinoma
PPI: protein–protein interaction
PRAD: prostate adenocarcinoma
READ: rectum adenocarcinoma
SCNAs: somatic copy number alterations
SKCM: skin cutaneous melanoma
STAD: stomach adenocarcinoma
TCGA: The Cancer Genome Atlas
THCA: thyroid carcinoma
UCEC: uterine corpus endometrial carcinoma

## 1 Introduction

Somatic copy number alteration (SCNA) is an important form of somatic genetic alteration in cancer (Kim *et al*., 2013). Some of the elements, including protein‐coding genes, microRNAs (miRNAs) and long non‐coding RNAs (lncRNAs) that are located in amplified or deleted regions, lead to altered activity in cancer cells (Czubak *et al*., 2015). Among the many protein‐coding genes or non‐coding RNAs located in regions of SCNAs, most likely only a fraction of them are cancer drivers (Silva *et al*., 2015). The identification of drivers within SCNA regions is an urgent and challenging task.

Several methods have been proposed to detect drivers with SCNAs. A simple approach is to seek known cancer genes within the regions of SCNAs to define the driver genes. For example, Borczuk *et al*. (2016) used census cancer genes to identify the driver genes in regions with significant SCNAs of malignant mesothelioma. However, seeking known cancer genes is often an unsuccessful approach in many regions because of the limited number of known cancer genes. For example, Zack *et al*. (2013) reported 140 focal SCNA regions in 4934 tumor samples across 11 cancer types, among which 102 regions were without known oncogene or tumor suppressor gene targets. More importantly, drivers identified by this method lack statistical significance, and it cannot detect new drivers, including driver non‐coding RNAs such as lncRNAs and miRNAs. Another method for revealing drivers with SCNAs is based on the hypothesis that drivers with SCNAs should result in corresponding gene expression changes, as only those SCNAs that cause changes in transcript abundance can possibly alter the corresponding activity of cancer cells (Du *et al*., 2013). For instance, Rubio‐Perez *et al*. (2015) selected as candidate drivers, those genes that exhibited SCNAs with significantly coherent expression changes. Du *et al*. (2013) selected lncRNAs whose SCNAs showed positive correlations with expression level changes as candidate drivers. Obviously, the drivers identified by this method have high false‐positive rates due to the broad criteria. The traditional and strict method to identify drivers in focal regions of SCNAs is to functionally test each gene in the region via biological experiments (Pon and Marra, 2015). For example, region 3q26 with 20 genes is frequently amplified in ovarian, breast and non‐small‐cell lung cancers. Through function interrogation, Hagerstrand *et al*. (2013) found that the increased expression of both *TLOC1* and *SKIL* induced subcutaneous tumor growth, and therefore, these two genes were identified as drivers of 3q26 (Hagerstrand *et al*., 2013). Although functional tests are a reliable way to identify drivers in focal SCNAs, they are time‐consuming and expensive and thus cannot be comprehensively applied to identify drivers with SCNAs in diverse cancer types. Sanchez‐Garcia *et al*. (2014) developed Helios to identify drivers with SCNAs for breast cancer, which mainly focused on protein‐coding genes. Hence, our work aimed to systematically distinguish drivers, including protein‐coding genes, lncRNAs and miRNAs, from passengers in regions of SCNAs by integrating expression profiles, known cancer genes and statistical control.

Here, our work aimed to identify the oncogenes and tumor suppressor genes that reside in recurrently aberrant genomic regions encoding a large number of genes, which is still a challenge for decoding the SCNAs involved in carcinogenesis. Known cancer genes have the characteristics of a high degree and large betweenness centrality in protein–protein networks (Jonsson and Bates, 2006), high mutation rate (Lawrence *et al*., 2014) and high conservation (Furney *et al*., 2008), which have been pursued in our work. Cancer genes tend to have interactive relationships in human protein–protein networks that are involved in the same modules and pathways underlying the hallmarks of cancer (Vinayagam *et al*., 2016; Wong *et al*., 2008). Thus, we hypothesized that driver genes in the region of SCNAs should have biological relationships, such as regulation or protein–protein interactions (PPIs), with known cancer genes and present significant expression correlation with known cancer genes in cancer samples. The Cancer Genome Atlas (TCGA) provides a large number of cancer samples for investigating the drivers in SCNA regions across different cancer types. We propose a new method to detect drivers (protein‐coding genes, lncRNAs and miRNAs) in the regions of SCNAs by analyzing a total of 8740 cancer samples of 18 cancer types from TCGA. Our work reveals many driver non‐coding RNAs in diverse cancer types. Analysis of drivers across multiple cancer types reveals specificity and commonality across cancers. Drivers shared by different cancer types could suggest novel drug repositioning.

## 2 Materials and methods

### 2.1 Datasets and processing

The level 3 RNAseq datasets (RNAseqV2 RSEM) of mRNA and miRNA for all 18 cancer types were downloaded from the Broad Institute, Firehose (http://gdac.broadinstitute.org/runs/stddata__2016_01_28/). For lncRNA, RNAseq RPKM profiles were downloaded from TANRIC (Li *et al*., 2015). The elements (protein‐coding genes, lncRNAs or miRNAs) with zero expression in at least 90% of samples were removed. All expression values were *z*‐score‐transformed for subsequent analysis.

Level 3 of the DNA copy number datasets of the Genome‐Wide Human SNP Array 6.0 platform were obtained from TCGA (https://cancergenome.nih.gov/). To detect significantly recurrent regions of SCNA, gistic 2.0 was applied to the level 3 segment data files (Mermel *et al*., 2011). We detected peak regions using a threshold of *q *<* *0.1 (results with *q *<* *0.25 and *q *<* *0.05 are listed in Table S6). Parameters used in the gistic algorithm were set as follows: cap values, 1.5; broad length cutoff, 0.5; confidence level, 0.99; joint segment size, 4; arm‐level peel‐off, 1; and maximum sample segments, 2500. Details for each parameter were described in a previous study (Mermel *et al*., 2011). We used a log2 ratio ± 0.25 as a cutoff to define copy number gain/loss similar to that of Bambury *et al*. (2015). Beroukhim *et al*. (2010) referred to arm‐level events as ‘gains’ or ‘losses’, and focal events as ‘amplifications’ or ‘deletions’. The copy number alterations identified by gistic 2.0 were focal regions and we therefore used ‘amplification’ and ‘deletion’ in our work. Statistics of the sample numbers for each cancer type are listed in Table S1.

### 2.2 Identification of drivers

**Figure 1:**
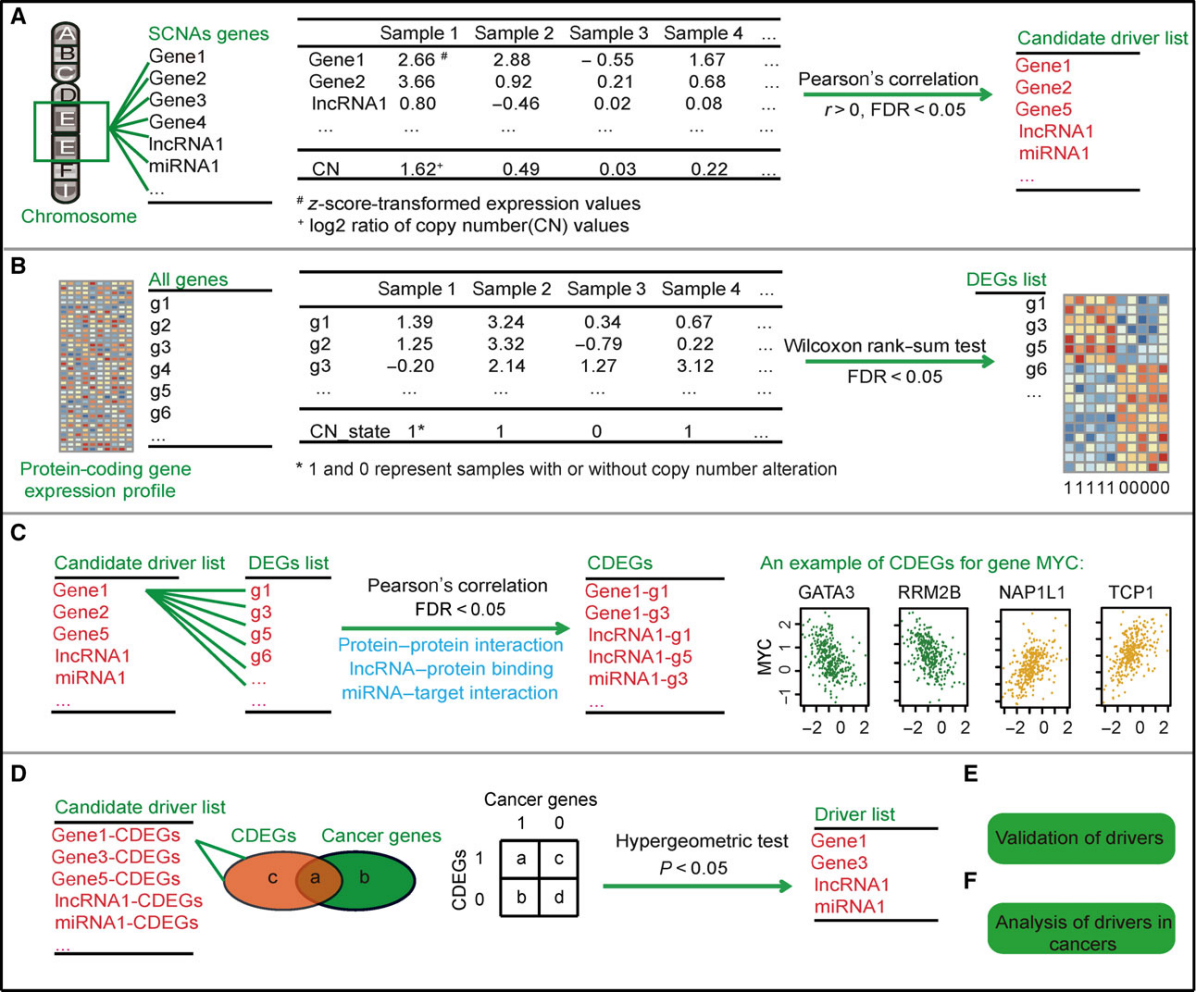
Schematic procedure of our work. (A) Identification of candidate elements whose expression levels are positively correlated with copy number alterations. (B) Identification of DEGs affected by the copy number alteration for each peak region. (C) Identification of CDEGs for each candidate element. PPIs, lncRNA‐protein binding relationships and miRNA‐target interactions are used to filter the CDEGs. (D) Identification of drivers whose CDEGs significantly overlap with known cancer genes. *a + b + c + d* is the total number of genes in the expression profile, and *a + b* is the number of census cancer genes in the expression profile. *a + c* is the number of CDEGs of one candidate driver. *a* is the number of overlapping genes between CDEGs and cancer genes. (E) Validation of drivers. (F) Analysis of drivers in multiple cancers.

To identify the drivers residing in the peak region of SCNAs, we performed the following analyses (Fig. 1):

Step 1 (Fig. 1A): Identify elements whose expression levels were consistent with copy number alteration. We used the Pearson correlation to test the correlation between expression and SCNAs of elements, including protein‐coding genes, lncRNAs and miRNAs. The *P*‐values were adjusted by Benjamini and Hochberg (BH). Furthermore, the elements with a false discovery rate (FDR) < 0.05 and *r *> 0 were retained as candidates for subsequent analyses.
Step 2 (Fig. 1B): Identify peak region‐related differentially expressed genes (DEGs). For each peak region, patients were separated into two groups: patients with and without SCNAs in this region. The Wilcoxon rank‐sum test was applied to identify DEGs between the two groups. The *P*‐values were adjusted by BH, and the genes with FDR < 0.05 were selected as DEGs.
Step 3 (Fig. 1C): Identify correlated differentially expressed genes (CDEGs) for each candidate element. The Pearson correlation test was used to calculate the expression correlation between the candidate elements and DEGs in samples with SCNAs of the candidate elements. Correlations among the elements within the same region were excluded. The *P*‐values were adjusted by BH, and the DEGs with FDR < 0.05 were retained. Then, only CDEGs having an interaction relationship, including PPI, lncRNA‐protein binding and miRNA‐target interaction, with candidate drivers were included.
Step 4 (Fig. 1D): Identify drivers in each peak region. We hypothesize that significant overlap between CDEGs and known cancer genes indicates that the candidate driver tends to regulate cancer genes and is more likely to be a driver during carcinogenesis. A total of 608 known cancer genes were obtained from the Cancer Gene Census on 13 February 2017 (http://cancer.sanger.ac.uk/census/) (Futreal *et al*., 2004). For each candidate element with *a + c* CDEGs derived from Fig. 1C, we calculated the probability (*P*‐value) of overlapping no less than *a* census cancer genes according to a hypergeometric distribution model. *a + b + c + d* is the total number of genes in the expression profile, and *a + b* is the number of census cancer genes in the expression profile. The probability of overlapping *a* census genes from *a + c* CDEGs by random chance is calculated using Eqn (1):

(1) $$\begin{array}{c} p=\frac{\left(\begin{array}{c} a+b\\ a \end{array}\right)\left(\begin{array}{c} c+d\\ c \end{array}\right)}{\left(\begin{array}{c} a+b+c+d\\ a+c \end{array}\right)} \end{array}$$

Then, the cumulative probability of overlapping no less than *a* census cancer genes between *a + c* CDEGs and *a + b* census cancer genes by random chance was calculated according to Eqn (2):

(2) $$\begin{array}{c} p=1-\sum_{k=0}^{a-1}\frac{\left(\begin{array}{c} a+b\\ k \end{array}\right)\left(\begin{array}{c} c+d\\ a+c-k \end{array}\right)}{\left(\begin{array}{c} a+b+c+d\\ a+c \end{array}\right)} \end{array}$$

### 2.3 Interaction dataset and other information

First, 608 641 PPI were downloaded from the InWeb_InBioMap database using version 2016_09_12 (https://www.intomics.com/inbio/map/#downloads). Then, 77 383 lncRNA‐protein binding pairs were collected from ChIPBase (http://rna.sysu.edu.cn/chipbase/) and NPInter (http://www.bioinfo.org/NPInter); 1 754 150 miRNA‐target interactions were collected from TargetScan (http://www.targetscan.org/vert_71/), miRanda (http://www.microrna.org/microrna/home.do), miRBase (http://www.mirbase.org/), miRTarBase (http://mirtarbase.mbc.nctu.edu.tw/) and starBase (http://starbase.sysu.edu.cn/).

### 2.4 Known cancer gene database

Cancer driver genes identified by at least two methods were collected from DriverDB (http://driverdb.tms.cmu.edu.tw/driverdbv2/). A total of 2027 cancer genes were collected from Bushman (http://www.bushmanlab.org/links/genelists), and 716 tumor suppressor genes were downloaded from TSGene (https://bioinfo.uth.edu/TSGene1.0/).

### 2.5 Information on drug target and drug response data

An integrated dataset of 24 141 drug‐target pairs was collected from DrugBank (http://www.drugbank.ca/), Cancer Cell Line Encyclopedia (CCLE; http://www.broadinstitute.org/ccle/home), Genomics of Drug Sensitivity in Cancer (GDSC; http://www.cancerrxgene.org/) and ChEMBL (https://www.ebi.ac.uk/chembl/). The drug response data of the cell lines were downloaded from the CCLE and GDSC. We used the half maximal inhibitory concentration values (IC50) in CCLE and the area above the fitted dose response curve (Act Area) in GDSC for the following analysis. A higher Act Area or lower IC50 means higher sensitivity to the drug. Information regarding known drug‐disease relationships was collected from DrugBank (http://www.drugbank.ca/) and the US Food and Drug Administration (FDA; https://www.accessdata.fda.gov/scripts/cder/daf/).

### 2.6 Other datasets

Pathway information was downloaded from Kyoto Encyclopedia of Genes and Genomes (KEGG, release 58.0). Synonymous substitution rate (*K*_s_) and non‐synonymous substitution rate (*K*_a_) data of human genes and mouse homologs were downloaded from NCBI HomoloGene (build 68). We calculated the *K*_a_/*K*_s_ ratio as the evolution rate. A smaller *K*_a_/*K*_s_ means that the gene is more conservative.

### 2.7 Bioinformatics tools

All analysis processes were performed in r 3.2.3 (https://www.r-project.org/). Network visualizations were performed in cytoscape 3.4.0 (http://www.cytoscape.org/).

## 3 Results

### 3.1 Focal SCNA regions are revealed in diverse cancer types

We analyzed the copy number profiles of 8740 cancer samples across 18 cancer types from TCGA. For each cancer type, gistic 2.0 was used to identify significantly recurrent regions of SCNA (Mermel *et al*., 2011). A total of 1160 peak regions were identified in 18 cancers with an average coverage of 418 Mb in each cancer. There were 462 amplified and 698 deleted peak regions. For each cancer, a mean of 4903 elements was included in the peak regions. The elements within each peak region include protein‐coding genes, lncRNAs or miRNAs. The statistics of the peak numbers and peak element numbers are listed in Table S2.

### 3.2 Identification of drivers in peak regions

We performed the following process to identify drivers (Fig. 1). First, only those protein‐coding genes or non‐coding RNAs with SCNAs that cause changes in transcript abundance can possibly alter the corresponding activity of cancer cells (Du *et al*., 2013). Thus, we selected the elements whose expression levels were significantly correlated with SCNAs as candidate drivers by Pearson correlation test with FDR < 0.05 and *r *>* *0 (Fig. 1A). Second, alterations, including SCNAs, that affect expression levels of other genes in the cancer genome have been used to identify key events for carcinogenesis (Masica and Karchin, 2011). Thus, we detected SCNA‐related DEGs for each peak region by comparing patients with and without alterations in this peak region (Wilcoxon rank‐sum test, FDR < 0.05; Fig. 1B). Third, we detected CDEGs for each candidate driver by calculating the correlation between the candidate driver and DEGs with FDR < 0.05. If the elements with SCNAs in peak regions truly affect the DEGs, the elements should have interactions or a regulatory relationship with CDEGs in addition to showing expression correlation with CDEGs. Based on this hypothesis, we filtered the CDEGs by PPI, miRNA‐target interaction and lncRNA‐protein binding information, which support the biological relationship between candidate driver and CDEGs (Fig. 1C). Finally, previous studies found that cancer genes have direct or indirect interactions in biological pathways or networks (Vinayagam *et al*., 2016). Thus, the CDEGs of a driver should significantly overlap with known cancer genes (*P *< 0.05, hypergeometric test; Fig. 1D). Detailed statistics of the results for each step in our work flowchart are summarized in Table S2.

**Figure 2:**
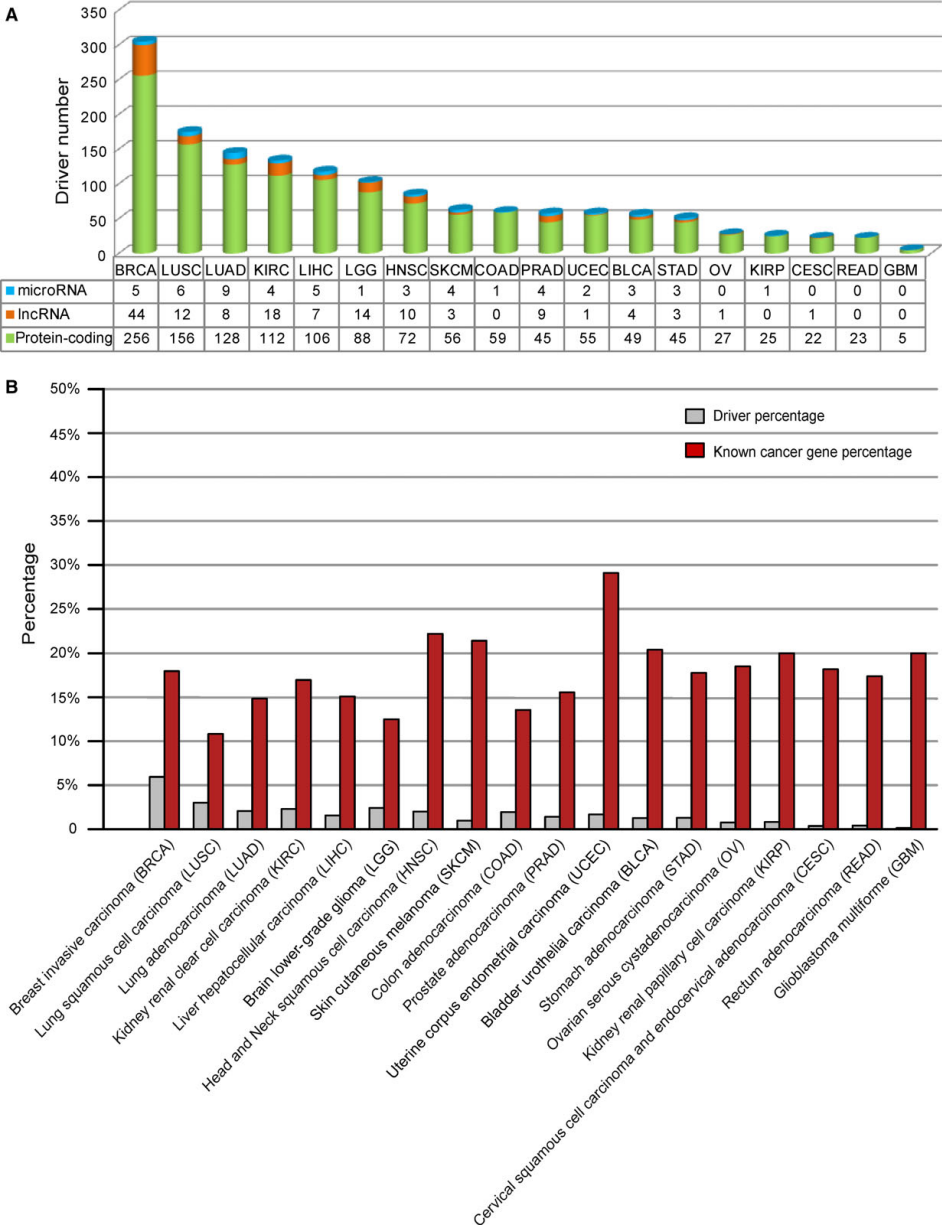
Statistics of drivers. (A) Statistics of the drivers in each cancer. Green, orange and blue cylinders represent driver protein‐coding genes, lncRNAs and miRNAs, respectively. (B) Overlap between drivers and census cancer genes. The gray pillar represents the ratio of drivers to all of the elements in the peak region in each cancer type. The red pillar represents the ratio of overlap between census cancer genes and drivers in each cancer type.

On average, 84 drivers were defined for each cancer type. Statistics for the number and type of driver of each cancer are shown in Fig. 2A, and drivers for each cancer are listed in Table S3. Among the 18 cancers, breast invasive carcinoma (BRCA) has the largest number of drivers, and glioblastoma multiforme (GBM) the smallest number. Nearly 1.7% of elements in the peak region were identified as drivers, and an average of ~ 18% of the drivers were known census cancer genes in each cancer (Fig. 2B).

### 3.3 Drivers capture the features of cancer genes

**Figure 3:**
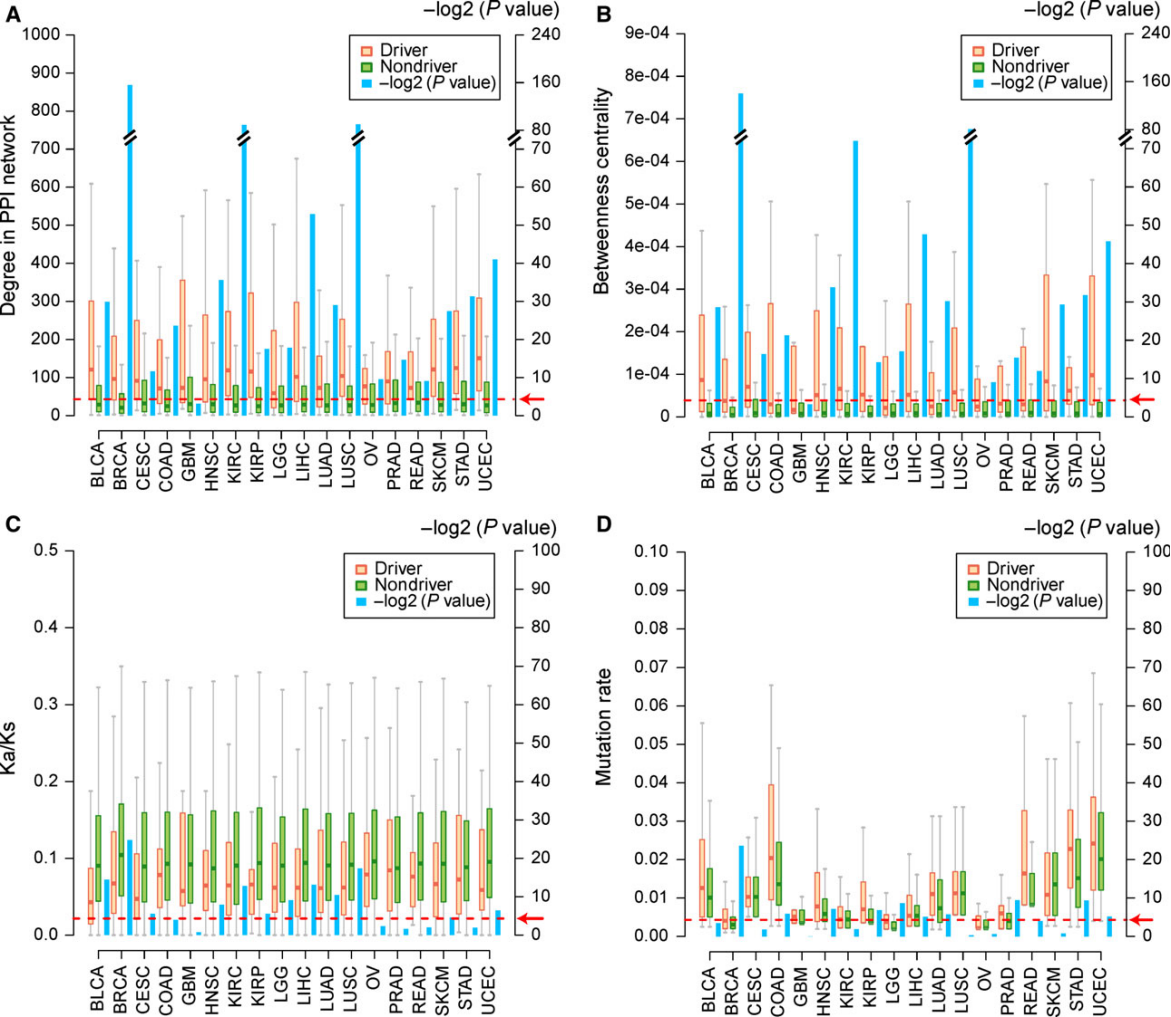
Comparisons between drivers and non‐drivers. (A) Degree in the human PPI network. (B) Betweenness centrality in the human PPI network. (C) *K*_a_/*K*_s_ ratio. (D) Mutation rate. The orange and green bars represent drivers and non‐drivers, respectively. The blue bar represents the *P*‐value tested by Wilcoxon rank‐sum test. *P*‐values were −log2‐transformed (*Y*‐axis in the right side). The red dotted line directed by the red arrow represents a *P*‐value < 0.05.

To confirm the drivers identified by our method, we compared the different attributes of drivers and non‐drivers. Here, both driver and non‐driver elements were limited to those whose expression levels were concordant with copy number alterations in peak regions. Cancer genes have a higher degree and larger betweenness centrality in the human protein–protein network and tend to be conserved across species (Furney *et al*., 2008; Jonsson and Bates, 2006; Xia *et al*., 2011). Drivers identified by our method also showed a significantly higher degree and larger betweenness centrality than did the those of non‐drivers in the PPI network (Fig. 3A,B; *P *< 0.05, Wilcoxon rank‐sum test). Twelve of the 18 cancers showed that the drivers have significantly lower *K*_a_/*K*_s_ ratios than those of non‐drivers (*P *< 0.05, Wilcoxon rank‐sum test; Fig. 3C). Furthermore, in six other cancers, the drivers showed the same tendency, indicating that the drivers tend to be conserved. Somatic mutations are another important mechanism employed by cancer genes to augment cancer progression (Czubak *et al*., 2015). The drivers tended to have a higher mutation rate than do non‐drivers (Fig. 3D; *P *< 0.05, Wilcoxon rank‐sum test). *P*‐values were calculated by Wilcoxon rank‐sum test and are marked as blue bars in Fig. 3. The red dotted line with an arrow corresponds to a *P*‐value < 0.05.

**Figure 4:**
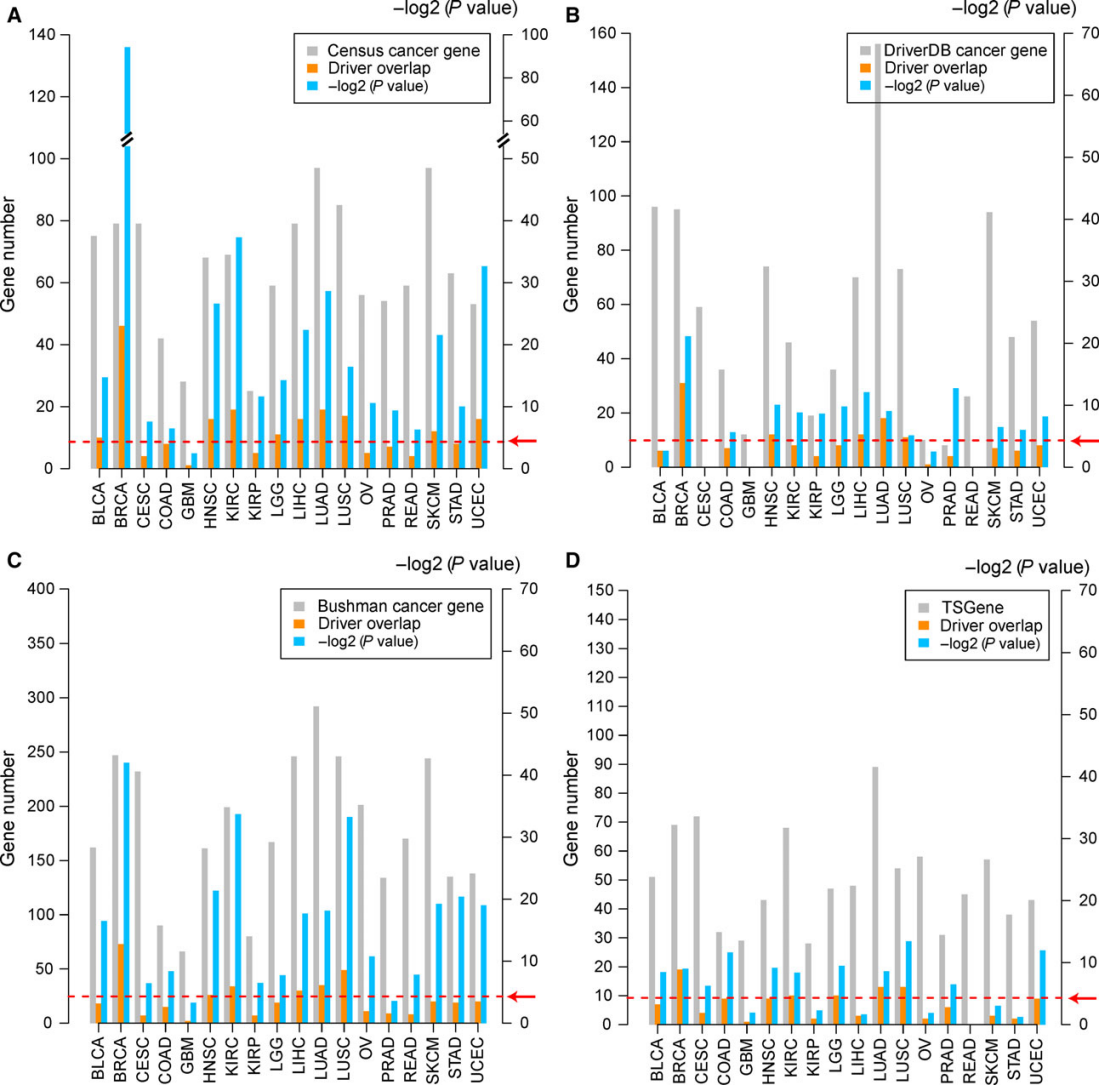
Overlap between drivers and known cancer genes. (A) Overlap between drivers and census cancer genes. (B) Overlap between drivers and cancer genes from DriverDB. (C) Overlap between drivers and cancer genes from Bushman. (D) Overlap between drivers with deletions and tumor suppressor genes in TSGene. The gray bars represent the number of known cancer genes. The orange bars represent the number of overlapping genes between drivers and known cancer genes. The blue bars represent the *P*‐values of overlapping genes between drivers and known cancer genes calculated by the hypergeometric test. *P*‐values were −log2‐transformed (*Y*‐axis in the right side). The red dotted line indicated by the red arrow represents a *P*‐value < 0.05.

We used different sources of cancer genes to further support the drivers identified in our work. Four databases of cancer genes were used, including census cancer genes (Futreal *et al*., 2004), DriverDB cancer genes (Chung *et al*., 2016), Bushman cancer genes (Sadelain *et al*., 2011) and TSGene tumor suppressor genes (Zhao *et al*., 2016) (see Materials and methods). Drivers identified by our method significantly overlapped with known cancer genes in most of the cancer types (Fig. 4A–C; *P *< 0.05, hypergeometric test). The drivers with deletions also significantly overlapped with tumor suppressor genes in the TSGene database in 11 of the 18 cancer types (Fig. 4D; *P *< 0.05, hypergeometric test). *P*‐values were calculated by hypergeometric test and are marked as blue bars in Fig. 4. The red dotted line with an arrow corresponds to a *P*‐value < 0.05.

### 3.4 Pan‐cancer analyses of drivers reveal specificity and commonality

**Figure 5:**
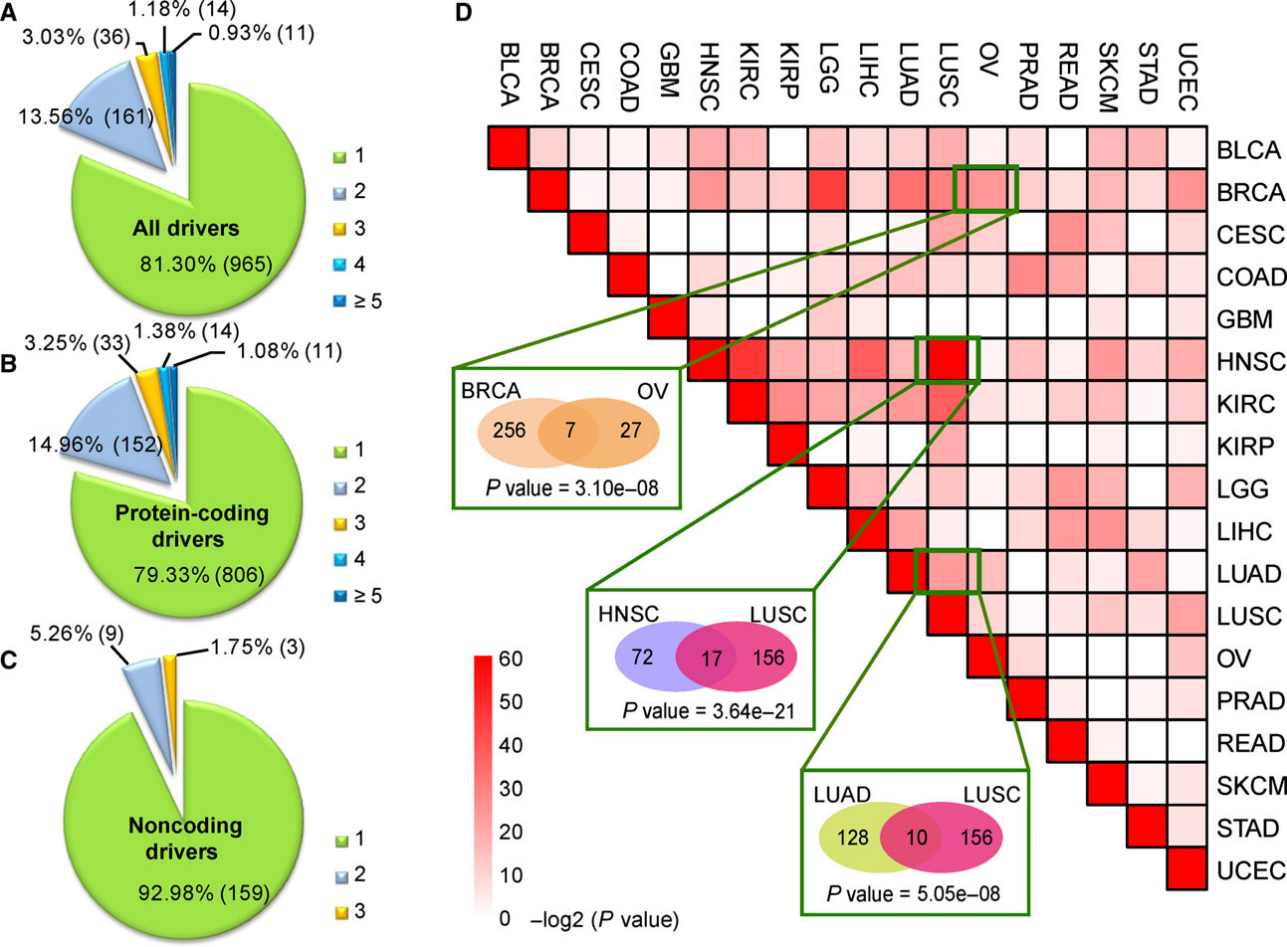
Distribution of drivers in 18 types of cancer. (A) Pie chart of drivers, including protein‐coding genes, lncRNAs and miRNAs. (B) Pie chart of driver protein‐coding genes. (C) Pie chart of driver non‐coding RNAs, including lncRNAs and miRNAs. Numbers 1–5 denote the number of cancer types that the driver presents. (D) Significance of overlapping drivers between cancer types. Color represents the −log2‐transformed *P‐*value calculated by the hypergeometric test. The numbers in the ellipses represent the number of drivers in the cancer type.

We next investigated the distribution of drivers across the 18 cancer types. The results showed that 81.3% of the drivers (including 806 protein‐coding genes, 117 lncRNAs and 42 miRNAs) were cancer‐specific and that 222 drivers were shared by at least two cancer types (including 210 protein‐coding genes, eight lncRNAs and four miRNAs; Fig. 5A). Interestingly, 79.3% of the driver coding genes were cancer‐specific (Fig. 5B) in contrast to 92.98% of the driver non‐coding RNAs (Fig. 5C). The driver non‐coding RNAs showed significantly more specificity of cancer type than that of the driver protein‐coding genes (*P *= 6.40 × 10^−6^, Fisher's exact test). In Fig. 5B, 152, 33, 14 and 11 protein‐coding genes were identified as drivers in two, three, four and more than five cancer types, respectively. For the 11 protein‐coding genes (*CDKN2A*,* CBL*,* GRB7*,* HDAC4*,* HRAS*,* MYC*,* POU5F1B*,* PIK3CD*,* RB1*,* RPS6KA1* and *ZBTB48*) shared by at least five cancer types, except *POU5F1B* (also known as OCT4‐pg1), all 10 of the other drivers have been recorded as cancer genes in the databases of census, DriverDB, Bushman or TSGene. Many studies have reported aberrations in *POU5F1B* in cancer, such as in gastric and prostate cancer (Hayashi *et al*., 2015; Kastler *et al*., 2010). In Fig. 5C, a total of nine non‐coding drivers (*hsa‐mir‐106b, hsa‐mir‐218‐2, hsa‐mir‐548k, AP006216.10, CAPN10‐AS1, RP11‐1191J2.4, RP11‐191L9.4, RP11‐443B7.1* and *RP11‐794P6.1*) were shared by two cancer types, and three non‐coding drivers (*PVT1, SOX2‐OT* and *hsa‐mir‐429*) by three cancer types.

To investigate the commonality between cancers, we tested whether pair‐wise cancer types significantly shared drivers using the hypergeometric model. The results revealed both known and new relationships of pair‐wise cancer types (Fig. 5D). For example, BRCA and ovarian cancer (OV), two female malignant tumors, exhibited significantly overlapping drivers (*P *= 3.10 × 10^−8^, hypergeometric test, Fig. 5D). In total, seven drivers (*MYC* with amplification, *PER2*,* HDAC4*,* PTPRG*,* PIK3R1*,* RAPGEF1* and *PPP5C* with deletion) were detected in both BRCA and OV. *MYC* is a known oncogene that is overexpressed in BRCA and OV, and *PER2* and *PRPRG* are known tumor suppressor genes in BRCA (Shu *et al*., 2010; Xiang *et al*., 2008). Lung adenocarcinoma (LUAD) and lung squamous cell carcinoma (LUSC), two main subtypes of non‐small cell lung cancer (NSCLC), also showed significantly overlapping drivers (*P *= 5.05 × 10^−8^, hypergeometric test, Fig. 5D). Interestingly, head and neck squamous cell carcinoma (HNSC) and LUSC had significantly overlapping drivers (*P *= 3.64 × 10^−21^, hypergeometric test, Fig. 5D). Both HNSC and LUSC belong to squamous cell carcinomas, and a total of 17 drivers were shared by HNSC and LUSC. *SOX2*, one of the 17 drivers, was amplified in HNSC and LUSC and has been reported as an oncogene in squamous cell carcinoma (Hussenet and du Manoir, 2010). Moreover, we found some other new pair‐wise cancer types with significantly overlapping drivers, e.g. bladder urothelial carcinoma (BLCA) and HNSC (*P *=* *7.79 × 10^−7^, hypergeometric test), BLCA and LUSC (*P *=* *1.87 × 10^−6^, hypergeometric test). The similarity in copy number alterations among HNSC, LUSC and BLCA may reflect a similar cell type of origin, as also reported by Hoadley *et al*. (2014). Information regarding the drivers shared by different cancer types is presented in Table S4. These results suggest that different cancer types may share common mechanisms of carcinogenesis.

### 3.5 Driver non‐coding RNAs have oncogenic and tumor‐suppressive roles in cancer

**Figure 6:**
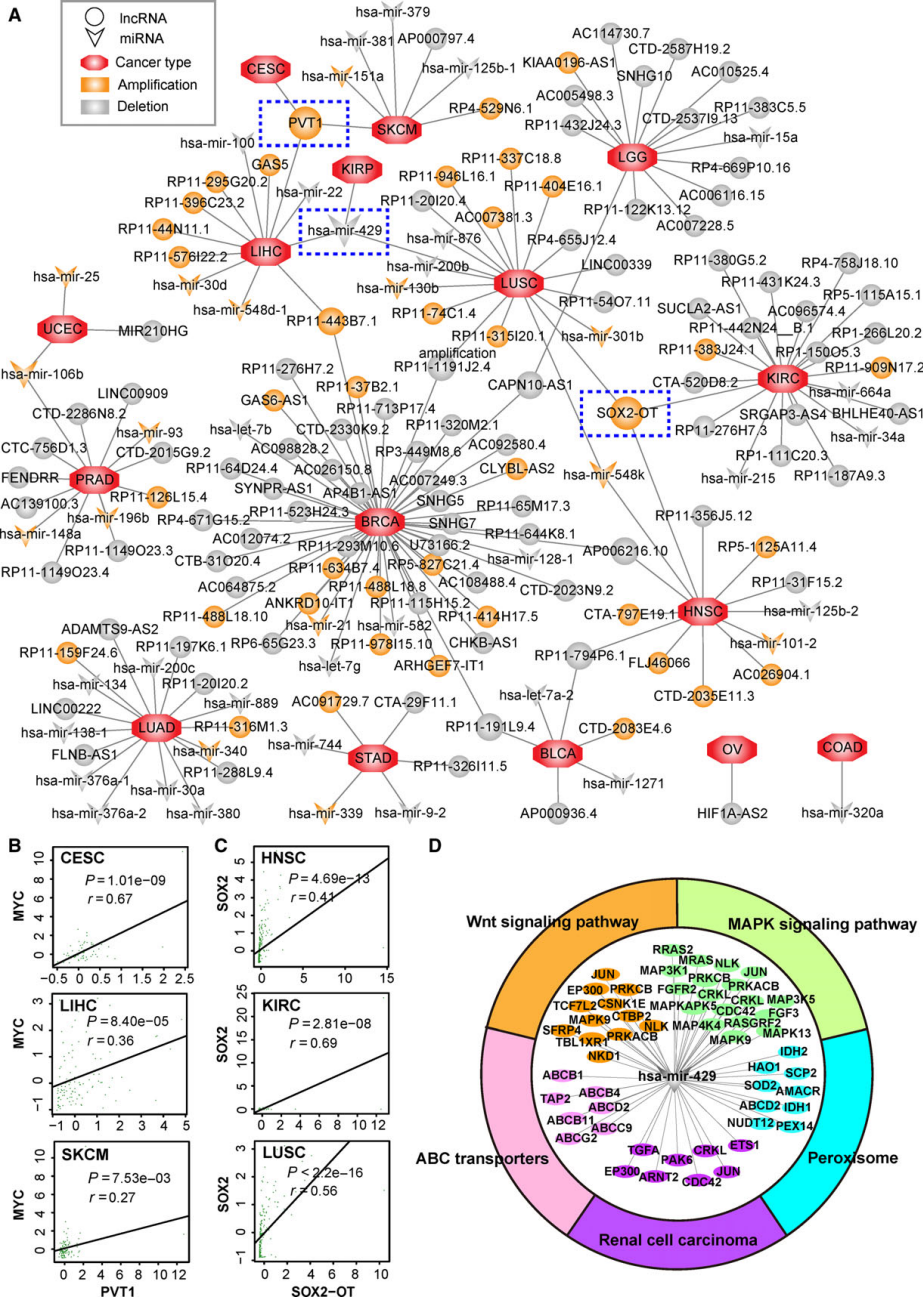
Driver lncRNA‐cancer network and functional analysis. (A) Network of driver lncRNAs and cancer types. Circles represent lncRNAs, and triangles represent miRNAs. Orange and gray colors represent amplification and deletion, respectively. The octangle represents cancer type. Edge represents the relationship between the identified driver and cancer type. The driver non‐coding RNAs identified in three cancer types are marked by blue dashed rectangles. (B) Pearson correlation of driver lncRNA *PVT1* and oncogene *MYC* in CESC, LIHC and SKCM samples with amplification of *PVT1*. The *z*‐score‐transformed expression profile was used. (C) Pearson correlation of driver lncRNA *SOX2‐OT* and *SOX2* in HNSC, KIRC and LUSC samples with amplification of *SOX2‐OT*. (D) KEGG enrichment analysis of targets of *has‐mir‐42*9 using the hypergeometric test with FDR* *< 0.05.

Copy number alterations of non‐coding RNAs play important roles in the progression of diverse types of cancer (Du *et al*., 2016). Our work revealed 125 driver lncRNAs and 46 driver miRNAs across 18 cancer types (Fig. 6A). Some relationships between cancer and driver lncRNAs or miRNAs identified in our work have been supported by data from the Lnc2Cancer database and the Human miRNA Disease Database (HMDD) (Li *et al*., 2014; Ning *et al*., 2016) (Table S5). For example, driver lncRNA *GAS5* with amplification in liver hepatocellular carcinoma (LIHC) identified in our work has been reported with oncogenic roles in LIHC by Tao *et al*. (2015). *Hsa‐mir‐134*, a driver miRNA with a deletion in LUAD, was found to suppress NSCLC progression through down‐regulation of *CCND1* (Sun *et al*., 2016).

A total of 12 driver non‐coding RNAs (eight lncRNAs and four miRNAs) were altered in at least two cancer types (Fig. 6A). Notably, *PVT1*,* SOX2‐OT* and *hsa‐mir‐429* were identified as common drivers in three cancer types. *PVT1* is a driver lncRNA that was amplified in cervical squamous cell carcinoma and endocervical adenocarcinoma (CESC), LIHC and skin cutaneous melanoma (SKCM), which has been reported to be related to the progression of CESC, LIHC and SKCM (Table S5). The *PVT1* locus resides ~ 2 Mb from the well‐known oncogene *MYC*. In our results, we observed a significant correlation between *PVT1* and *MYC* in CESC (*r *= 0.67, *P *= 1.01 × 10^−9^, Pearson correlation), LIHC (*r *= 0.36, *P *= 8.40 × 10^−5^, Pearson correlation) and SKCM (*r *= 0.27, *P *= 7.53 × 10^−3^, Pearson correlation; Fig. 6B). *SOX2‐OT*, another common driver lncRNA in HNSC, kidney renal clear cell carcinoma (KIRC) and LUSC, participated in the regulation of oncogene *SOX2*. Pearson correlation analysis revealed that *SOX2‐OT* and oncogene *SOX2* had a significant correlation in HNSC (*r *=* *0.41, *P *=* *4.69 × 10^−13^, Pearson correlation), KIRC (*r *=* *0.69, *P *=* *2.81 × 10^−8^, Pearson correlation) and LUSC (*r *=* *0.56, *P *=* *2.21 × 10^−16^, Pearson correlation; Fig. 6C). *Hsa‐mir‐429* was identified as a deleted driver in kidney renal papillary cell carcinoma (KIRP), LIHC and LUSC in our work. Hidaka *et al*. reported that *hsa‐mir‐429* has a potential tumor suppressor role in renal cell carcinoma (Hidaka *et al*., 2012). We performed KEGG pathway enrichment analysis of targets of *hsa‐mir‐429* that showed expression correlation in KIRP, LIHC and LUSC. Five KEGG pathways (MAPK signaling pathway, peroxisome, Wnt signaling pathway, ABC transporters and renal cell carcinoma) were significantly enriched with targets of *hsa‐mir‐429* (FDR < 0.05, hypergeometric test), suggesting a possible functional role of *has‐mir‐429* in the carcinogenesis of KIRP, LIHC and LUSC (Fig. 6D).

### 3.6 Drivers shared by different cancer types suggest drug repositioning

**Figure 7:**
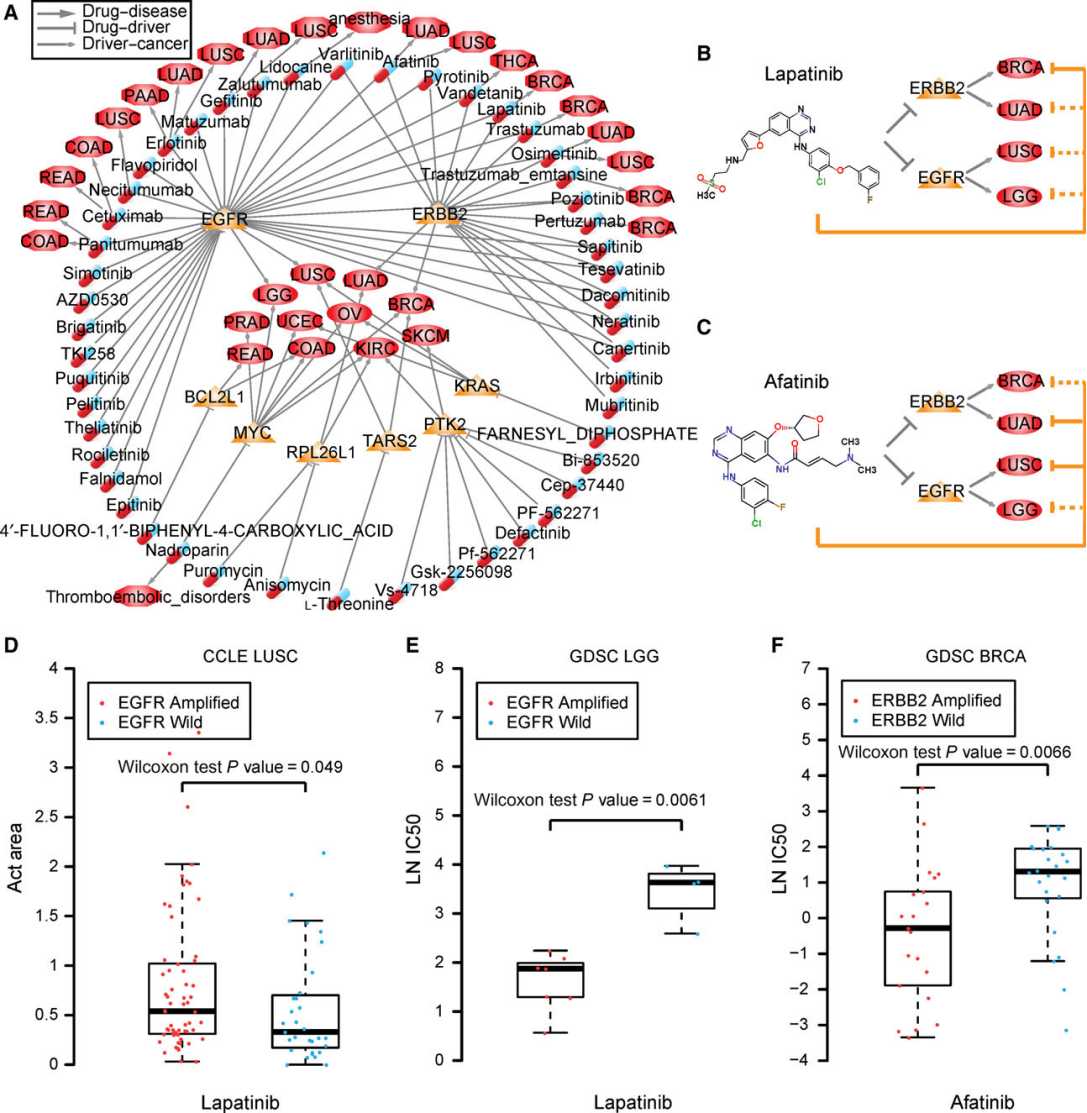
Cancer driver‐drug network. Relationship of drug, driver and cancer. Triangles and ellipses represent drivers and cancer types. The drivers in specific cancer types are connected by rhombus arrows. The relationship of drugs (capsules) targeting the drivers are marked by T‐type arrows. The known drug and disease (octagons) relationships are marked by arrows. (B) Lapatinib and targets in cancers. (C) Afatinib and targets in cancers. The orange solid T‐type line represents known drug‐cancer relationships, and the orange dotted T‐type line represents the predicted drug‐cancer relationship. (D) Box‐plots of Act Area values of LUSC cell lines treated with lapatinib in the CCLE database. (E) Box‐plots of IC50 values of LGG cell lines treated with lapatinib in the GDSC database. (F) Box‐plots of IC50 values of BRCA cell lines treated with afatinib in the GDSC database. The IC50 values are natural logarithm‐transformed. In (D–F), the red dots present amplification of the corresponding gene in cell lines, and the blue dots represent wild‐type. Wilcoxon rank‐sum test was used.

The pan‐cancer analyses of drivers presented above indicate that some cancer types have similar causes and may be treated by the same drugs, which provides a new method for investigating drug repositioning. Considering that most targeted drugs exhibit anti‐cancer effects by blocking targets that are overexpressed (Gharwan and Groninger, 2016), we focused on drivers with amplifications in cancer. In total, 36 driver genes were amplified in at least two cancer types, and eight of them were targeted by 49 known drugs. The drug and target information were integrated from DrugBank, CCLE, GDSC and ChEMBL (see Materials and methods). Then, the cancer driver‐drug network was constructed (Fig. 7A). In Fig. 7A, triangles and ellipses represent drivers and cancer types, respectively. The drivers in specific cancer types are connected by a rhombus arrow. The relationships of drugs (capsules in Fig. 7A) targeting the drivers are marked by T‐type arrows. On the basis of the known drug‐disease associations, we connected the drug and disease (octagons in Fig. 7A) by arrows in the network.

We hypothesized that two cancers having common drivers could be treated by the same drug (Rubio‐Perez *et al*., 2015). For example, *ERBB2*, targeted by lapatinib and afatinib, is a driver of BRCA and LUAD. *EGFR*, also targeted by lapatinib and afatinib, is a driver of LUSC and lower grade glioma (LGG) in the brain. Lapatinib has been approved by the FDA for the treatment of *ERBB2* (*HER2*) overexpressed metastatic breast cancer (Ryan *et al*., 2008) (Fig. 7B). Afatinib, another FDA approved drug, is used to treat late stage (metastatic) NSCLC with *EGFR* mutations (Dungo and Keating, 2013) (Fig. 7C). LUSC cell lines with amplification of *EGFR* show marginally significant sensitivity to lapatinib compared with those of LUSC cell lines with wild‐type *EGFR* in the CCLE database (*P *= 0.049, Wilcoxon rank‐sum test, Fig. 7D). LGG cell lines with amplification of *EGFR* show significant sensitivity to lapatinib compared with those of LGG cell lines with wild‐type *EGFR* in the GDSC database (*P *= 6.1 × 10^−3^, Wilcoxon rank‐sum test, Fig. 7E). BRCA cell lines with amplification of *ERBB2* show significantly lower IC50 levels of afatinib compared with those of BRCA cell lines with wild‐type *ERBB2* in the GDSC database (*P *= 6.1 × 10^−3^, Wilcoxon rank‐sum test, Fig. 7F). Thus, we inferred that afatinib could be used to treat BRCA, and lapatinib could be used to treat LUSC and LGG. Lin *et al*. (2012) reported promising clinical activity of afatinib in HER2‐positive breast cancer patients who had progression following trastuzumab treatment in a Phase II study. Ramlau *et al*. determined a potential use for lapatinib combination therapy in NSCLC through a Phase I study (Ramlau *et al*., 2015).

## 4 Discussion

A critical challenge in the genome‐wide analysis of SCNAs is distinguishing the alterations that drive cancer growth from the numerous random alterations that accumulate during carcinogenesis. In this study, we explored a new method for identifying drivers in regions with significant SCNAs. We revealed an average of 84 drivers for each cancer type, including protein‐coding genes, lncRNAs and miRNAs, and found that the drivers showed attributes of cancer genes (Fig. 3A–D) and significantly overlapped with known cancer genes (Fig. 4A–D). Our method identified not only many known cancer genes such as *CCND1*,* MYC*,* ERBB2*,* RB1* and *BRCA1* in breast cancer but also many novel protein‐coding genes as well as non‐coding RNAs that may contribute to carcinogenesis (Table S3).

Pan‐cancer analysis of drivers could reveal similarities among cancer types from different tissues by their genomic signatures (copy number alterations). Consistent with histological classifications, our work found that LUAD and LUSC as well as OV and BRCA showed significantly overlapping drivers. Furthermore, we found some new cancer types with similar genomic alterations, such as HNSC and LUSC, and LGG and BRCA (Table S4). LGG and BRCA had a significant overlap of 16 drivers (*P *=* *1.39 × 10^−14^, hypergeometric test), including known cancer genes *CDKN2A*,* HRAS*,* MYC* and *RB1*. Both *CDKN2A* and *RB1* were reported to have deletions in BRCA and LGG (Bieche and Lidereau, 2000; Debniak *et al*., 2004). The pan‐cancer analysis of drivers could help us understand carcinogenesis as well as provide new strategies for cancer therapy. Notably, our work investigated the similarity and specificity of different cancer types from copy number alterations, which only represents one kind of molecular feature of cancer cells. In the future, we will systematically perform the pan‐cancer analysis by integrating multiple molecular features, such as methylation and expression of mRNAs, miRNAs and proteins.

Helios is another method that was used to identify amplified drivers in BRCA (Sanchez‐Garcia *et al*., 2014). The drivers with amplifications in BRCA identified by our work significantly overlapped those detected by the Helios method (*P *=* *9.46 × 10^−10^, hypergeometric test), including *CCND1, MYC, ERBB2, ERLIN2, FOXA1, RAD52* and *TOMM20*. Compared with Helios, our method could not only find amplified and deleted coding gene drivers but could also determine many driver non‐coding RNAs, including 44 lncRNAs and five miRNAs (Fig. 6A). However, as done in Helios, employing shRNA data to further validate drivers with amplifications in BRCA identified in our work is a good strategy and warrants attention in the future.

Our work used gistic 2.0, a state‐of‐the‐art algorithm, to detect recurrent regions with SCNAs. van Dyk *et al*. (2016) reported that gistic 2.0 tends to call larger deletion regions than amplification regions and identifies more drivers in deleted regions than in amplified regions for breast cancer. Generally, our methods identified more drivers with deletions than drivers with amplifications for each cancer type, except for LIHC, which has 77 amplified drivers and 41 deleted drivers (Table S2). Deleted drivers in 11 cancer types identified by our method significantly overlap with known tumor suppressor genes recorded in the TSGene database (*P *<* *0.05, hypergeometric test; Fig. 4D). Some studies have also found that deletions or losses are more common than amplifications or gains in cancer (Cancer Genome Atlas Network, 2012; Schoch *et al*., 2002), which is an interesting phenomenon that warrants further exploration.

The key aspect of our method for identifying drivers is to test whether the candidate drivers significantly regulate known cancer genes. Thus, for each candidate driver, we intersected the known cancer genes with the CDEGs rather than with the candidate driver itself. We used the census cancer genes from COSMIC, which is a widely used cancer gene set because of its strict standards. However, most of the census cancer genes were identified based on mutation analysis. The mutation status of known cancer genes may affect the expression of CDEGs. In our method, we used expression correlation and regulatory interaction data to reduce the influence induced by the alteration status of known cancer genes. Moreover, the number of census cancer genes is far from the actual number of cancer genes in the human genome. When more real cancer genes are employed by our method, the drivers derived by our method will be more precise. In this study, the new drivers captured the characteristics of cancer genes, such as a high degree and large betweenness centrality in the protein–protein network, high conservation, and so on, which supports the reliability of our results. Of course, validation of the drivers by biological experiments warrants detailed studies in the future.

## Author contributions

YYG and ZG conceived and designed the project. WBZ, ZXZ and YH acquired the data. WBZ and RPW performed the statistical analysis. WBZ and CYW designed the algorithm. WBZ and ZXZ analyzed and interpreted the data. YYG and WBZ wrote the paper. HHL, FY and YH interpreted the results and revised the final manuscript. LSQ and CGW revised the manuscript.

## Supporting information


**Table S1.** Statistics of samples in 18 cancer types.

**Table S2.** Statistics of driver identification in each step.

**Table S3.** Drivers in each cancer.

**Table S4.** (A) The hypergeometric test *P‐*values for overlapping between cancer types. (B) The number of overlapping drivers between cancer types. (C) Information on drivers amplified in at least two cancer types. (D) Information of drivers deleted in at least two cancer types.

**Table S5.** Driver non‐coding RNAs and cancer relationships supported by literature from lnc2cancer and HMDD.

**Table S6.** Statistics of driver identification with different q thresholds using gistic 2.0.
